# News and Miscellany

**Published:** 1915-03

**Authors:** 


### News and Miscellany.

### Dr. Eagon Passes Away.

Dr. S. Eagon, a well known physician of Dallas, died at his home, 3805 Bowser Street, January 30.

Dr. Eagon was born at Staunton, Va., May 2, 1835, came of revolutionary ancestry on both sides. The obituary of his grandfather states that "He was one of the first martyrs to the cause of American liberty." His father, Dr. John Eagon, was once mayor of Staunton. His mother was the daughter of Charles Yancey, who was a colonel in the war.

Dr. Eagon graduated from the University of Virginia in 1856; took a post-graduate course at the College of Physicians and Surgeons of New York under Dr. Austin Flint. He taught in the New Orleans School of Medicine until the beginning of the Civil War. During the war he had charge of the Trans-Mississippi hospital of the Confederacy. After the war he practiced medicine at Marshall, Sherman and Jefferson, Texas, finally settling in Dallas about thirty years ago.

Dr. Eagon married Miss Elizabeth Smith, daughter of Robert Smith of Louisiana. Surviving him are three children, Robert Eagon of Dallas, Mrs. Ben J. Tillar of Fort Worth, and Elizabeth Eagon Hurst.

Chivalrous, cultured and as tender as a woman, Dr. Eagon was loved by all who knew him. His generosity and kindness to the poor won him many friends. He will long be remembered for the good things he did.

